# Morphological Metaphor Mapping of Moral Concepts in Chinese Culture

**DOI:** 10.3389/fpsyg.2020.554061

**Published:** 2020-12-17

**Authors:** Yingjie Liu, Kang Li, Lina Li, Jing Zhang, Yuerui Lin, Baxter DiFabrizio, He Wang

**Affiliations:** ^1^School of Psychology, North China University of Science and Technology, Tangshan, China; ^2^College of William and Mary, Williamsburg, VA, United States

**Keywords:** moral concept, shape, rotation, Chinese culture, conceptual metaphor theory, morphological metaphor

## Abstract

According to conceptual metaphor theory, individuals are thought to understand or express abstract concepts by using referents in the physical world—right and left for moral and immoral, for example. In this research, we used a modified Stroop paradigm to explore how abstract moral concepts are metaphorically translated onto physical referents in Chinese culture using the Chinese language. We presented Chinese characters related to moral and immoral abstract concepts in either non-distorted or distorted positions (Study 1) or rotated to the right or to the left (Study 2). When we asked participants to identify the Chinese characters, they more quickly and accurately identified morally positive characters if they were oriented upright or turned to the right and more quickly and accurately identified immoral characters when the characters were distorted or rotated left. These results support the idea that physical cues are used in metaphorically encoding social abstractions and moral norms and provided cross-cultural validation for conceptual metaphor theory, which would predict our results.

## Introduction

Morality is a reflection of fundamental judgments about good and bad, right and wrong. It plays an indispensable role in enhancing human well-being by accurately assessing the social desirability of a behavior or belief (Haidt and Algoe, 2004). The moral concept is a very important abstract concept in social life, and its understanding is also realized through a relatively concrete concept. Conceptual metaphor theory (CMT) proposes that humans tend to rely on metaphors as an efficient way to ground abstract concepts in a physical, relatable reality (Lakoff and Johnson, 1999, 2008).

Metaphors connect abstract concepts (which CMT calls the “target domain”) to more concrete concepts (the “origin domain”) to make abstract concepts more understandable (Lakoff, 2008). In CMT, an origin domain refers to the cognitive domain consisting of specific events we are familiar with or that can be directly experienced. A target domain refers to the cognitive domain consisting of abstract concepts hard to understand or perceive, which often rely on the vocabulary and imagery of the origin domain to be expressed (Lakoff and Johnson, 2008).

Metaphor is a powerful cognitive tool for understanding abstract moral concepts (Landau et al., 2010). Lakoff and Johnson (1999) proposed that that humans organize and construct conceptual systems through a few specific basic concepts, including a set of spatial relationships (such as up and down, left and right), a set of physical ontological concepts (such as entities, containers), and a set of behaviors (such as eating and walking). All of these basic concepts are derived from human sensorimotor experience. Through cross-domain mapping, we construct and understand abstract concepts via projections and consecutive applications of basic concrete concepts. Thus, the conceptual metaphors of morality and immorality are clarified in terms of some common contrastive categories from our bodily experience in the physical environment. For example, in English, “high-minded” or “on the up and up” is used to describe a moral person, and “down and dirty,” “low-minded,” or “underhanded” is used to describe an immoral person (Lakoff and Johnson, 1999). In addition to this spatial position metaphor, morality is also represented in metaphors by concrete concepts, such as brightness, colorfulness (Hill and Lapsley, 2009; Sherman and Clore, 2009), and cleanness (Haidt and Joseph, 2004; Zhong and Liljenquist, 2006; Schnall et al., 2008). In one study, when the word “God” (morality) was presented in the center of the screen, the attention of the individual would be diverted to the above and right spatial field of vision, and the display of “demon” related words (immorality) shifted the individual’s visual attention to the below and left spatial field of vision, presumably in accordance with the learned association of right and left (“sinister” is the Latin word for “left”) (Chasteen et al., 2010). To sum up, these studies involve color, light spectrum contrasts, and directional and spatial metaphors to help describe morality and its social concomitants. However, the origin domain does not only include the dimensions as mentioned earlier but also includes shape, location, and other orienting properties. For instance, the English expression “an upright position” has been used to describe a person with strong morals and seems to come from the perspective of “morphology.” The morphology refers to the appearance or image of objects in existence or the form of expression under certain conditions. Morphological metaphors are different from common spatial metaphors; it has to do with its own properties. According to the morphological characteristics of objects, we can divide it into skewness, rotation, stand upside down, orientation, or its own size. Further, morphological metaphor can be extended from the perspectives of space metaphor, location metaphor, color metaphor, etc., and expand the research scope of moral metaphor.

Research on the metaphorical characteristics of moral cognition has focused mainly on the English language (Camgöz et al., 2002; Meier et al., 2004). However, the cultural background also plays an important role in the study of moral concepts and their metaphors. Culture influences not only the personal embodied metaphor (Gibbs and Berg, 1999) but also the metaphorical representation of thinking and language (Xinya and Zhongyi, 2015). It follows that language may actually dictate how moral metaphors are encoded and how concepts from the origin domains are configured to express the target domains—inherently creating differences in moral concepts across languages and cultures. The Chinese perspective on the metaphor of moral imagination deserves special attention, as it may indeed have markedly different representations of moral concepts because of its linguistic distinctiveness. Both from the structure of Chinese characters and its extended meaning, it has special morphological metaphor characteristics. In Chinese, “
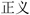
” (zhèng yì, “justice”), “
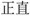
” (zhèng zhí, upright), “
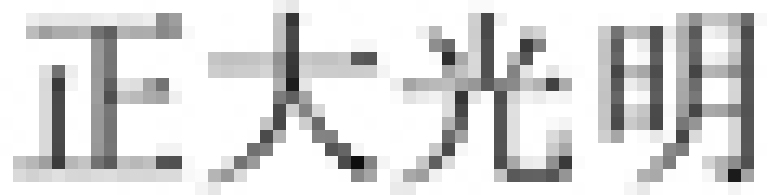
” (zhèng dà guâng míng, “aboveboard”), and “
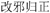
” (gai xié guî zhèng, “on the straight”) are often used to describe a person’s moral integrity; all these words encompass the character “
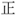
” (zhèng), which means “standard, not deviating, not bending, properly proportioned.” We also use “
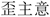
” (wâi zhu yì, bad ideas), “
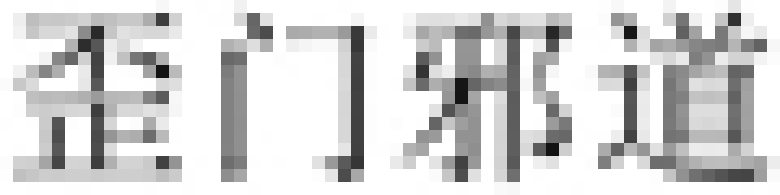
” (wâi mén xié dào, crooked ways), and “
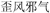
” (wâi fçng xié qì, evil winds) to describe a person’s bad moral character; all these words contain the character “
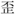
” (wâi), which means “skew, distortion.” In English, people often use “straight” and “upright” to describe honest and reliable, whereas using “devious” and “oblique,” which mean crooked, to describe something that is unjust or immoral—for example, “achieve the goal by means of a devious path” or “an oblique political maneuver.” Chinese also regard immoral behaviors as the distortion of moral concepts; hence, uprightness and distortion correspond, respectively, to morality and immorality.

Does our cognitive architecture effectively connect metaphorical representations of moral concepts with morphology? If the CMT model is an accurate representation of how we articulate and encode abstractions, we would expect that the particular property of objects traditionally associated with a certain concept (for example, “straight,” “up,” “right,” “clear”) would cue faster recognition of those concepts (“morality,” “honesty,” “valor,” etc.). We would also expect the CMT to predict that the particular property of objects traditionally associated with concepts with lower social desirability or negative valence (“deceit,” “cruelty,” “betrayal,” “rage”) would also be more readily recognizable in their stereotypical characteristics (“crooked,” “left,” “down,” “distorted”). We would explore whether the morphological characteristics of objects or words can also be associated with positive moral or negative, immoral concepts.

Orientation is a vital task in the human cognitive system, and many abstract concepts rely on spatial metaphors to be construed clearly (Lakoff and Johnson, 2008). There are three diametrically opposed, generally asymmetric mental axes in the body: up and down, left and right, and front and back (Tversky, 2008). The left and right directional indicators have been mapped concurrently onto metaphorical representations of moral concepts. Chinese traditional culture often refers to “
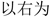

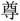
” (yi yòu wéi zûn, “take right side as honor”). The host should take the initiative to stay on the left side and make the right side free for guests, honoring their presence. Men take the initiative to make their right side available for women when standing together, out of respect. Juniors should give the right-of-way and their right-side standing position up to their elders. Many cultural phenomena further indicate that horizontal spatial orientation has been related to moral concepts (“right” referring to moral rectitude and “left” referring to immoral or aberrant behavior). These perceptual cues or orientation of the objects can also be redundantly mapped onto each other: right can also refer to up, and left refers to down.

In Chinese, we often use the word “
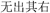
” (wú chû qí yòu, “second to none”) to refer to one’s moral integrity, whereas “
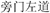
” (páng mén zuo dào, “heterodoxy”) is commonly used to refer to impure or improper methods or ways. Even today, the words such as “
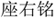
” (zuò yòu míng, motto) and “
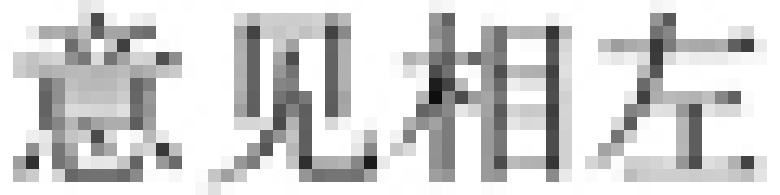
” (yì jiàn xiâng zuo, “difference of opinion”) still indicate that the right side is honored. The Chinese characters “
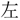
” (zuo) and “
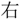
” (yòu) mean left and right, respectively. Not only in the background of Chinese culture but also in the expression of English, “right” often has a positive connotation, such as intelligence (the Latin word for right-handed was “dexter,” from which the word dexterous comes from). For example, “a right-hand man,” “to right a wrong.” On the other hand, “left” often express a negative meaning, such as clumsiness, socially suboptimal, or socially undesirable conduct. For example, “two left feet,” “out in left field,” and one from English, a “left-handed compliment.” Chasteen et al. (2010) showed that when people process the words related to their god or holy items, they responded faster to the stimulus on the right than left. Contrarily, when people processed the words related to the devil, they responded faster to the stimulus on the left than right. It is plausible to infer that left and right could be perceptual, orientational signals that cue the moral concepts of right and wrong, especially as they, like a number line, instruct a sort of directionality and imply a visual contrast. Left and right might be metaphorical representations of moral concepts in Chinese culture, but then again, they might not.

In the present research, we used an experimental paradigm borrowing the basic principle of the Stroop paradigm (Stroop, 1938). We manipulated the congruence words’ meanings and their visual display and used response time to measure facilitation or interference effects on word semantic classification (moral vs. immoral). We applied this experimental paradigm to test the possible facilitation of the proposed morphological metaphor mapping for moral concepts present in Chinese culture. Thus, we tested whether moral concepts are more readily pairable with “upright” characters and immoral with distorted characters in Study 1. In Study 2, we checked to see if the left–right association also mapped onto immoral and moral concepts, respectively. Detecting meaningful associations along these lines would lend credibility to the morphological metaphor mapping concept as a psychologically generalized reality.

## Study 1 the Morphological Metaphor of Moral Concept: From the Upright and Skewed Perspective

The aim of study 1 was to investigate whether moral words are easier to read when clear and immoral words are comparatively easier to read when distorted, compared with the inverse (moral words distorted vs. immoral words non-distorted). We hypothesized participants would more quickly recognize non-distorted moral words and distorted immoral words (congruent condition) and that they would take more time to parse non-distorted immoral words and distorted moral words (incongruent condition).

## Materials and Methods

### Participants

Thirty-four college students voluntarily participated in study 1 [14 males, 20 females; their mean age was 18.26 years, and standard deviation (SD) was 0.89]. All were right-handed. Participants were recruited through advertisements posted in school social spaces. Participants contacted us and volunteered to participate in the experiment. All participants had normal vision or corrected vision. At the end of the experiment, participants were paid according to their winnings and debriefed.

### Stimulus Construction and Evaluation Methodology

#### Moral and Immoral Words

To create stimuli, we took 20 two-character moral words from the Chinese edition of modern Chinese frequency dictionary, such as honesty, nobility, purity, etc., and 20 two-character words describing immoral states of immoral concepts, such as betrayal, dishonor, dirtiness, ridicule, punishment, etc., (see Table 1). For example, the word frequency of 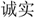 (honest) was 0.00076 (the 0.00076 represents the word occurred 0.00076 times in a million). There was no significant difference in the words’ frequency of usage between moral words (*M* = 0.00086) and immoral words (*M* = 0.00102), *t*(38) = −0.87, *p* = 0.390. Word frequency information was obtained from “The Corpus System of Modern Chinese Research” of Beijing Language and Culture University^1^. There was no significant difference in the number of strokes making up the characters of our moral words (*M* = 17.74) and immoral words (*M* = 18.50), *t*(38) = −0.71, *p* = 0.51.

**TABLE 1:** List of moral words and immoral words.

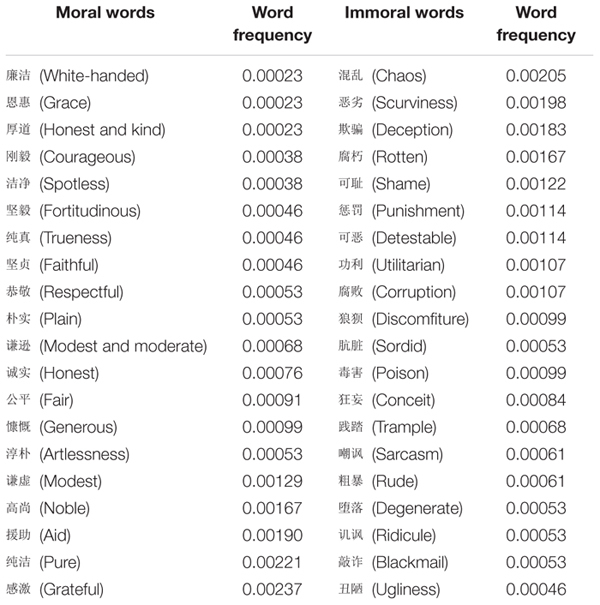	

Before the formal experiment, we assessed the adequacy (the degree of validity and recognition) of the moral and immoral words we chose during a pretest (Yang et al., 2017; Zhai et al., 2018). We asked a different set of 30 participants, who were not part of the formal experiment, to rate the moral valence of these words on a nine-point scale, from 1 for “highly immoral” to 9 for “highly moral.” We found that the mean score for moral words (*M* ± *SD* = 7.33 ± 0.45) was significantly higher than 5, the middle point of the nine-point morality rating scale, *t*(19) = 23.18, *p* < 0.001. The mean score of immoral words (*M* ± *SD* = 3.225 ± 0.71) was significantly lower than 5, *t*(19) = -11.164, *p* < 0.001. The chosen moral and immoral words aligned with participants’ experience of them and met the requirements of the experiment—specifically, our moral and immoral language samples discretely and reliably represented their intended corpora.

#### Distortions of Verbal Stimuli

We randomly selected five moral and five immoral words from the experimental materials we had chosen and used Adobe Photoshop’s built-in distortion function to *morph* each character, twisting each character into 45° and 90° distortions to both their left and right sides—as if the center of the character was a whirlpool’s epicenter, bending the characters around its nexus. We then assessed how our stimuli would feel in terms of their degree of distortion and ease of recognition with a separate pretest. Another 30 participants, neither part of the formal experiment nor the prior pretest, rated the degree of distortion on a seven-point scale from 1 for “no distortion at all” to 7 for “very distorted.” They also rated the relative ease of recognition on a seven-point scale from 1 for “it was hard to identify” to 7 for “it was easy to identify.” Statistical analysis using a one-way analysis of variance (ANOVA) showed that the main effect of the degree of distortion was significant, and the mean score of 90° distortion to the left (*M* ± *SD* = 5.47 ± 0.73) was significantly higher than other distortions, *F*(3) = 102.02, *p* < 0.001, η*_*P*_*^2^ = 0.73. The difference in the degree of recognition was not significant, *F*(3) = 0.10, *p* = 0.964; words were generally as recognizable across the 45° and 90° distortions. Therefore, we chose to distort the words 90° to the left for our formal experimental materials.

### Experimental Design and Procedure

We used 2 (word type: morality vs. immorality) × 2 (word shape: distortion vs. no distortion) within-subjects design. The dependent variables were participants’ (1) accuracy of identifying the word’s moral category and (2) reaction time (RT). We based our procedure and implementation of the experiment on that of prior research (Yu et al., 2016; Yang et al., 2017).

We used the Stroop inhibitory strategy to probe the effects of deforming the words on the time it took participants to recognize them (Stroop, 1938; Federica and Tagini, 2017). Target words in the experimental task were presented in the center of the computer screen with the background set as 50% grayscale (set the red green blue values of 50% grayscale as red = 128, green = 128, and blue = 128). All words were in Song typeface, with a font size of 48 pounds, and then every single word was processed into an image of 550 × 300 pixels. The distorted words in the experiment were generated by the “Distortion” function in Adobe Photoshop. Every distorted word was distorted 90° to the left. No distorted words were presented in a familiar or standard form. Unaltered words were presented simply in the Song typeface without further processing. Each of the 20 moral words and the 20 immoral words was presented in both “distortion” and “no distortion” states only once, for a total of 80 trials. Stimuli were presented in completely random order.

At the beginning of the experiment, participants were asked to sit in front of the computer approximately 30 cm away from the screen and place their left index finger on the key “F” of the keyboard and their right index finger on the key “J.” They were told that during the experiment, they would have to make accurate and rapid judgments about the inherent characteristics of the words—“Does the word you see describe a Moral or Immoral concept?”

The experimental procedure consisted of two parts: the practice and the experimental trials. Before the formal experiment, non-experimental stimuli (10 moral words and 10 immoral words) were used to help participants practice the task—and the computer would automatically give feedback (right or wrong category) about their responses. Participants completed the 20 practice trials; after the practice, they saw a screen reading, “Press any key to start the formal experiment.” During the formal experiment, participants pressed buttons according to the instructions, and the target words were presented randomly—this time, without feedback as to whether they sorted the word into the correct category or not. Participants were instructed to press “F” for immoral words and press “J” for moral words. The key assignments were counterbalanced between participants, and the computer measured participants’ RT and accuracy for each trial.

**FIGURE 1:**
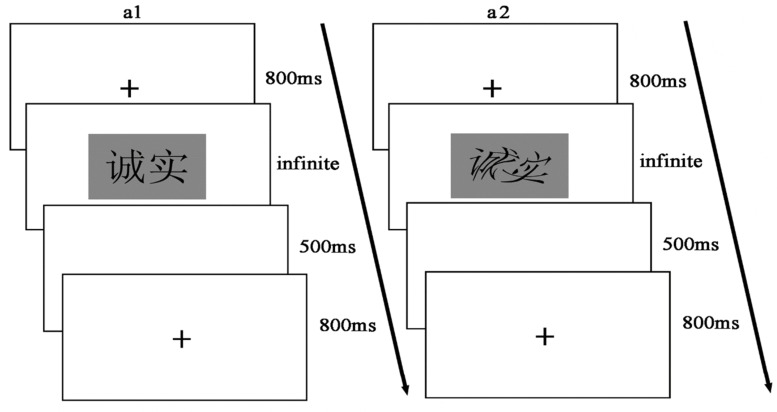
Experimental flow chart for Study 1. a1 represents a no distortion trial and a2 a distortion trial.

Before each stimulus was presented, a red “ + ” fixation point was displayed in the center of the computer screen for 800 ms. Then the target word was presented, and participants were asked to make corresponding keystrokes according to the category (morality or immorality) the word belonged to. For example, 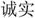 (chéng shi, meaning “honesty”) corresponds to morality. A blank screen was presented for 500 ms during the intertrial interval (see Figure 1).

## Results

**FIGURE 2:**
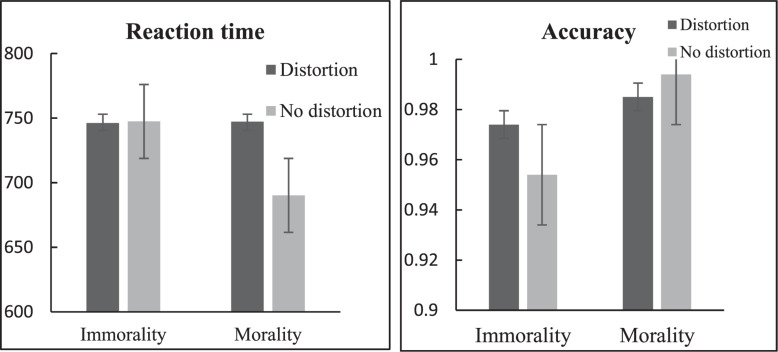
RT and accuracy of morality vs. immorality judgments under word shape conditions (distorted vs. non-distorted). Error bars = SEM.

The accuracy rate of all participants in Study 1 was above 97%. We excluded trials with RT shorter than 300 ms and deleted 3 SDs below and above the mean (approximately 2% of trials) and the inaccurate trials. A repeated-measures ANOVA using a 2 (word type: morality vs. immorality) × 2 (word shape: distortion vs. no distortion) design was conducted on RT. A significant main effect of word type was revealed [*F*(1, 33) = 4.81, *p* = 0.035, η*_*P*_*^2^ = 0.13]. Specifically, the RT for morality was faster than immorality (see Table 2). The main effect of word shape was significant [*F*(1, 33) = 7.20, *p* = 0.011,η*_*P*_*^2^ = 0.18]. The RT for distortion was slower than no distortion (see Table 2). Importantly, a significant interaction between word type and word shape was observed [*F*(1, 33) = 9.89, *p* = 0.004, η*_*P*_*^2^ = 0.23]. *Post hoc* comparisons revealed that the RT for morality concepts under distortion (*M* ± *SD* = 747.24 ± 117.83) was slower than it under no distortion (*M* ± *SD* = 690.13 ± 118.66), *p* < 0.001; the RT showed no difference between immorality concepts under distortion (*M* ± *SD* = 746.13 ± 133.82) and immorality under no distortion (*M* ± *SD* = 747.39 ± 132.63), *p* = 0.937. Furthermore, we also found that the RT showed no difference between morality under distortion and immorality under distortion, *p* = 0.940; but the RT for immorality under No distortion was slower than morality under no distortion, *p* = 0.002 (see Figure 2).

**TABLE 2:** Means and standard deviations of RT and accuracy.

		**RT(ms)(*M* ± *SD*)**	**Accuracy(*M* ± *SD*)**
Word type	Morality	718.69 ± 113.21	0.99 ± 0.02
	Immorality	752.65 ± 124.95	0.96 ± 0.04
Word shape	Distortion	746.69 ± 118.59	0.98 ± 0.03
	No distortion	718.76 ± 115.91	0.97 ± 0.04

A repeated–measures ANOVANOVA using a 2 (word type: morality vs. immorality) × 2 (word shape: distotortion vs. no distotortion) design was conducted on accuracy. The main effect of word type was significant [*F*(1, 33) = 17.92, *p* < 0.001, η_*P*_^2^ = 0.35], the accuracy for morality was higher than immorality (see Table 2). The main effect of word shape was not significant [*F*(1, 33) = 0.42, *p* = 0.523]. A significant interaction between word type and word shape was observed [*F*(1, 33) = 7.92, *p* = 0.008, η*_*P*_*^2^ = 0.19]. *Post hochoc* comparisons revealed that the accuracy for morality under distotortion (*M* ± *SD* = 0.98 ± 0.23) was lower than it under no distotortion (*M* ± *SD* = 0.99 ± 0.12), *p* = 0.044; the accuracy for immorality under distotortion (*M* ± *SD* = 0.97 ± 0.17) was higher than it under no distotortion (*M* ± *SD* = 0.95 ± 0.41), *p* = 0.050 (see Figure 2).

## Discussion

Using a modified Stroop paradigm, we asked participants to make judgments about the underlying social quality (morality or immorality) of words that we presented either in a non-distorted or a distorted state. Results indicate that participants’ average RT for morality words was significantly faster than immorality words. Participants’ average RT for distortion was slower than that of no distortion (“Upright”). Furthermore, their RT for morality under distortion was slower than it was under no distortion. Average RT did not differ between immorality words under distortion and it under no distortion.

In terms of accuracy of identification, morality was higher than immorality, and morality under distortion was lower than it under no distortion. Accurately identifying immorality words was more likely in the distortion condition than in the no distortion condition. These findings support the conclusion that word shape and presentation (non-distortion vs. distortion) may have metaphorical representations consistent with abstract moral concepts. To further scrutinize this phenomenon, in Study 2, we explored whether Chinese moral concepts also evinced the same sort of differential facilitation and hindrance effects along the orientations of right vs. left.

## Study 2 the Morphological Metaphor of Moral Concept: From the Perspective of Left Rotation or Right Rotation

Study 2 meant to investigate whether the morphological metaphor mapping of moral concept might help us predict the cognitive behavior of participants seeing moral and immoral words presented rotated toward the right or the left, seemingly triggering an association with right and wrong. We hypothesized participants would have a faster reaction time to moral words that were rotated to the right and immoral words that were rotated to the left (“congruent”) and a slower reaction time to immoral words that were rotated to the right and moral words that were rotated to the left (“incongruent”).

## Materials and Methods

### Participants

Thirty-eight college students voluntarily participated in study 2 (16 males, 22 females; their mean age was 18.30 years, and SD was 0.86); all were right-handed. Participants were recruited in the same way as Study 1. At the end of the experiment, participants were paid according to their winnings and debriefed.

### Experimental Material and Evaluation Method

The corpus of moral and immoral words for Study 2 came from Study 1. However, in Study 2, each experimental word was rotated either 45° to the left or to the right in Adobe Photoshop. Prior research shows that mental rotation with an angle of less than 60?can affect word recognition (Koriat and Norman, 1985). Some researchers had also studied the recognition of Chinese characters and found that the mental rotation of 45° had an impact on the recognition of Chinese characters (Bolin et al., 1995), so we used 45 degrees of rotation for our experimental material.

Each of the 20 moral and immoral words was presented in the states of both 45° rotation to the left and 45° rotation to the right. Every single word was processed into an image of 550 by 350 pixels to ensure that the font could be fully displayed on the screen after the rotation. Each word is presented twice (once tilted to the right, once tilted to the left). There were a total of 80 trials, and the presentation order was completely random.

### Experimental Design and Procedure

We used a within-subjects experimental design of 2 (word type: morality vs. immorality) × 2 (word rotation: left rotation vs. right rotation) within-subjects design. The dependent variables were the accuracy and RT.

**FIGURE 3:**
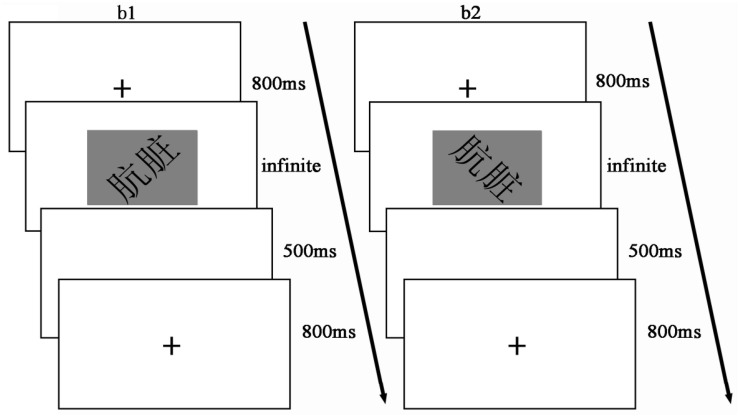
Experimental flow chart for study 2. b1 represents a left rotation trial and b2 a right rotation trial.

The experimental procedure in Study 2 was essentially that of Study 1, except that the independent variable of “word shape” in Study 1 now became “word rotation” in Study 2 (see Figure 3). Although we physically altered the shape of words in Study 1, in Study 2, the words themselves were unchanged but merely rotated 45°. The participants were presented with two completely different forms of vision (see Figure 3). We referred to previous empirical studies that recommended the rotation factor of 45° (Ścigała and Indurkhya, 2016; Wang et al., 2016; Li and Cao, 2017; Zhai et al., 2018; Schneider et al., 2020). For example, 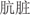 (ang zang, means dirty) corresponding to immorality.

## Results

**FIGURE 4:**
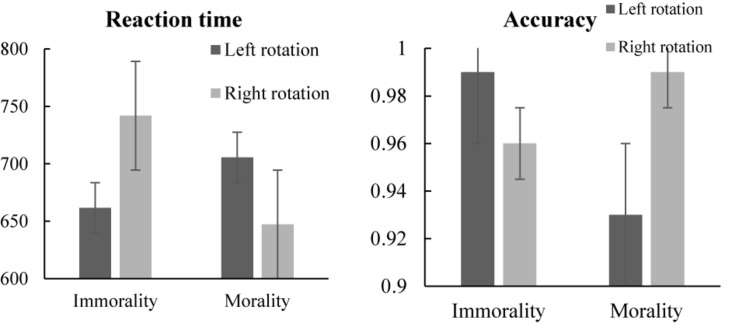
RT and accuracy of morality vs. immorality judgments under word rotation conditions (left vs. right). Error bars = SEM.

The accuracy rate of all participants of Study 1 was above 95%. We excluded trials with RT shorter than 300 ms and deleted 3 SDs below and above the mean (approximately 3% of trials) and the inaccurate trials. A repeated-measures ANOVA using a 2 (word type: morality vs. immorality) × 2 (word rotation: left rotation vs. right rotation) design was conducted on RT. A significant main effect of word type was revealed [*F* (1, 37) = 4.50, *p* = 0.041, η*_*P*_*^2^ = 0.11]; the RT for morality was faster than immorality (see Table 3). The main effect of word rotation was not significant [*F*(1, 37) = 0.79, *p* = 0.377]. However, a significant interaction between word type and word rotation was observed [*F*(1, 37) = 31.26, *p* < 0.001, η*_*P*_*^2^ = 0.46]. *Post hoc* comparisons revealed that the RT for morality under left rotation (*M* ± *SD* = 705.56 ± 84.20) was slower than it under right rotation (*M* ± *SD* = 647.06 ± 66.58), *p* = 0.001; the RT for immorality under left rotation (*M* ± *SD* = 661.55 ± 87.72) was faster than it under right rotation (*M* ± *SD* = 741.78 ± 105.13), *p* < 0.001 (see Figure 4).

**TABLE 3:** Means and standard deviations of RT and accuracy.

		**RT(ms)(*M* ± *SD*)**	**Accuracy(*M* ± *SD*)**
Word type	Morality	676.31 ± 55.01	0.960 ± 0.09
	Immorality	701.66 ± 79.85	0.980 ± 0.02
Word rotation	Left rotation	683.55 ± 69.94	0.963 ± 0.09
	Right rotation	694.42 ± 67.87	0.976 ± 0.02

Similar to the analysis of RT, to scrutinize accuracy of participants’ judgments across conditions, we conducted a repeated-measures ANOVA. The main effect of word type was not revealed [*F*(1, 37) = 2.11, *p* = 0.155] (see Table 2). The main effect of word rotation was not significant [*F*(1, 37) = 0.83, *p* = 0.368]. However, a significant interaction between word type and word rotation was observed [*F*(1, 37) = 6.64, *p* = 0.014, η*_*P*_*^2^ = 0.15]. *Post hoc* comparisons revealed that the accuracy for morality under left rotation (*M* ± *SD* = 0.93 ± 0.19) was lower than it under right rotation (*M* ± *SD* = 0.99 ± 0.02), *p* = 0.041; the accuracy for immorality under left rotation (*M* ± *SD* = 0.99 ± 0.01) was higher than it under right rotation (*M* ± *SD* = 0.96 ± 0.04), *p* < 0.001 (see Figure 4).

## Discussion

Study 2 used a modification of the Stroop paradigm to explore the accessibility of moral and immoral concepts when presented in the right or left rotations (considered the metaphorically “congruent” condition) vs. left and right rotations (considered “incongruent”). RT for morality was faster than immorality. The interaction effect showed that the RT for morality words was faster under the right rotation than when under left rotation; immorality words under left rotation were identified faster than when under the right rotation. For accuracy, only the interaction effect was significant. Participants classified morality concepts less accurately under the left rotation than it under the right rotation. They identified immoral concepts under left rotation was more accurately than under the right rotation.

Word rotation, such as left–right rotation, may have metaphorical representations consistent with abstract moral concepts, supporting the domain-generalization of CMT. We also found the scientific evidence for the concept of “
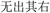

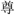
” (take right side as honor) in traditional Chinese culture.

## General Discussion

In the current research, we used a variation on the Stroop paradigm to conduct two studies investigating the morphological metaphor mapping of moral concepts against the backdrop of Chinese cultural expression. Specifically, we looked at moral and immoral concept vocabulary that were either presented with a non-distortion or a distortion or rotated either right or left to see if the CMT’s theoretical framework would accurately predict human cognitive behavior when identifying these words. Participants identified words that were distorted or on the left faster if they were about immoral concepts, and they identified words describing moral concepts more readily if the words were non-distorted and rotated to the right. Moral concepts were easier to classify than immoral concepts and generally were identified faster. In “incongruent” conditions, identifying the moral and immoral words took longer (moral x distorted/left or immoral x non-distorted/right), even though the words were basically controlled for by shape and number of brushstrokes.

Accuracy of identifying the concepts also paralleled the congruent or incongruent condition—it was easier for participants to identify words if they were in a metaphorically stereotyped posture or shape. These results suggest that word shape and word rotation had metaphorical representations that were consistent with abstract moral concepts. These results further confirmed the morphological metaphor of moral concepts had psychological reality.

In the study, there is an interaction between word type and word shape, i.e., the RT for morality words was faster under the right rotation than when under the left rotation; immorality words under the left rotation were identified faster than when under the right rotation. The traditional Chinese etiquette usually takes the right as the top, for respect, and the left as the bottom for inferiority. The right usually represents high status, positive and positive, whereas the left represents low status, negative and derogatory. Studies have also shown that when there is a phenomenon, the right side is good, and the left side is bad in Western culture. Positive valence concepts such as intelligence and nobility are usually associated with the right of the space, whereas negative valence concepts such as clumsiness and inferiority are associated with the left of the space (Jewell and McCourt, 2000). Also, in popular belief, morality is always just, and immorality is always oblique. In our other study, it was still found the RT for morality under distortion was slower than it was under no distortion. The result also further supported the difference in the association between moral and immoral words with different morphology. Language is encoded in the body of knowledge and information (Louwerse, 2008). The formation of conceptual meaning is not determined by a single factor but involves a variety of coded types of information, some with perceptual characteristics (e.g., embodied, modal) and some with non-perceptual characteristics (e.g., verbal symbols, modeless) (Dove, 2010). In the perception of moral words, people often accept that morality is positive and that distorted morality does not correspond to reality.

According to the theory of experiential cognition and perceptual simulation, perceptual representation is automatically activated when the vocabulary is processed (Barsalou, 2011). In our studies, people identified moral words faster than immoral ones. Some theorize that identifying moral, socially desirable behavior is primal and “direct” and hence inherently faster (Chen et al., 2018). Moral words could be the metaphorical prototypes of concepts that are deformed to create words for immoral concepts. On the other hand, Hill and Lapsley (2009) posit that individuals pay more attention to *immoral* events to protect themselves from betrayal and injury, a phenomenon called immorality bias. Under this model, participants focus more on immoral words and do more perceptual processing, resulting in a slower RT in our tasks. Positively valenced words are usually processed faster than negative words (Kauschke et al., 2019), which would suggest that moral words are processed faster than words about immoral abstractions.

The availability of concrete referents could also drive the observed RT difference. Real-life referents are more easily simulated or visualized by perception because they have realistic counterparts grounded in perceptual experience, and they could have just been the default association for moral concepts (Holcomb et al., 1999; Binder et al., 2005; Wang et al., 2010; Laszlo and Federmeier, 2011). Our research cannot decide which of these views is correct.

Lakoff and Johnson (1999) argue that abstract concepts are based on concrete sensory experience. People might consider that the expression or representation of morality should hence be positive or upright. This mapping of moral conceptual metaphors could automatically activate the corresponding spatial representations and subtly influence an individual’s cognition through their coding of that spatial representation. Studies have shown that concrete concepts were more easily simulated or visualized by perception because they have realistic counterparts to provide a perceptual experience (Holcomb et al., 1999; Binder et al., 2005; Wang et al., 2010; Laszlo and Federmeier, 2011). In life, people might pay more attention to morality than immorality and could be more sensitive to concepts related to morality, whereas the immoral concepts had not formed an obvious metaphorical representation in people’s minds. Moral intuition is essentially a stable, innate moral belief, knowledge, or ability (Sinnott-Armstrong et al., 2010). Moreover, Chinese cultural iconography and language are profoundly metaphorical. People generally use words such as “
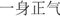
” (yî shçn zhèng qì, upright) to describe moral people. CMT’s predictions—that we use concrete features of the physical world (the origin domain) to imbue a sense of physicality to abstract moral and social concepts (the target domain)—bore out using the Chinese language, providing new evidence of CMT’s generalizability as a theory of human moral cognition and communication (Williams et al., 2009; Landau et al., 2010; Pecher et al., 2011).

From the perspective of vision, it has been found that words related to “moral” can effectively activate the response of people to “up,” whereas words related to “immoral” can activate the response of individuals to “down,” namely “moral up, immoral down” (Chasteen et al., 2010). Moreover, the concept of “
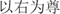
” (take the right side as honor) has existed in traditional Chinese culture since ancient times. People associate the word “
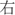
” (right) with positive things. Metaphor and metonymy play important roles in the representations of objects and events and the constructions of mental images in terms of basic everyday experiences (Landau et al., 2010).

Limitations of this study highlight the need for future research. Firstly, our research only focused on word shape and word rotation. Other sorts of deformations may help clarify the facilitating effect of the physical reference point as a metaphorical guide for identifying symbolic language. Secondly, there may be some potential confounding effects in the manipulation of the valence of moral and immoral words. The positive valence is inherent to moral stimuli as well as negative valence to immoral stimuli. The “right side” metaphorical advantage for moral words could be because they are positive valued words and the “right” advantage works as well for other positive valued abstract concepts. We should pay more attention to this effect and make a clearer distinction between the potency and manipulation of words in future studies. Thirdly, our experiments could be tinged with error coming from individual’s highly variant skills with written language. For future research, we could adopt other experimental paradigms, such as the implicit association test, using images and audiation paradigms to see how effectively CMT predicts individual behavior across the senses or in different cognitive frameworks. From the perspective of experimental technology, advanced cognitive neural technologies, for example, ERP and fMRI, could also be used to shed new light on how the mapping between moral concepts and morphological metaphors leads to firm inferences.

## Data Availability Statement

The raw data supporting the conclusions of this article will be made available by the authors, without undue reservation.

## Ethics Statement

The studies involving human participants were reviewed and approved by North China University of Science and Technology. The patients/participants provided their written informed consent to participate in this study.

## Author Contributions

YiL conceived the manuscript, ran statistical analyses, and contributed to the manuscript. KL contributed to the manuscript. LL conceived, modified the manuscript, and contributed to the manuscript. JZ, YuL, BD, and HW conceived the manuscript and contributed to the manuscript. All authors contributed to the article and approved the submitted version.

## Conflict of Interest

The authors declare that the research was conducted in the absence of any commercial or financial relationships that could be construed as a potential conflict of interest.

## Footnotes

1 http://corpus.zhonghuayuwen.org/index.aspx
